# Octopod PtCu Nanoframe for Dual-Modal Imaging-Guided Synergistic Photothermal Radiotherapy: Erratum

**DOI:** 10.7150/thno.107893

**Published:** 2025-01-01

**Authors:** Jinghua Li, Xiangyang Zu, Gaofeng Liang, KeKe Zhang, Yuliang Liu, Ke Li, Zhong Luo, Kaiyong Cai

**Affiliations:** 1 School of Medical Technology and Engineering, Henan University of Science and Technology, Luoyang 471023, China;; 2 School of Materials Science and Engineering, Henan University of Science and Technology, Luoyang 471023, China;; 3 Key Laboratory of Biorheological Science and Technology, Ministry of Education, College of Bioengineering, Chongqing University, Chongqing 400044, China.

The authors regret that the original version of our manuscript contained some inadvertent errors that presented in Figure 1A, Figure 3C and Figure 8 M, which was caused by our careless mistakes during figure assembly. We thus make an Erratum. The correct version of the Figure 1A, Figure 3C and Figure 8 M are shown below. The authors confirm that the corrections made in this erratum do not affect the original conclusions. The authors apologize for any inconvenience or misunderstanding that the errors may have caused.

## Figures and Tables

**Figure 1:**
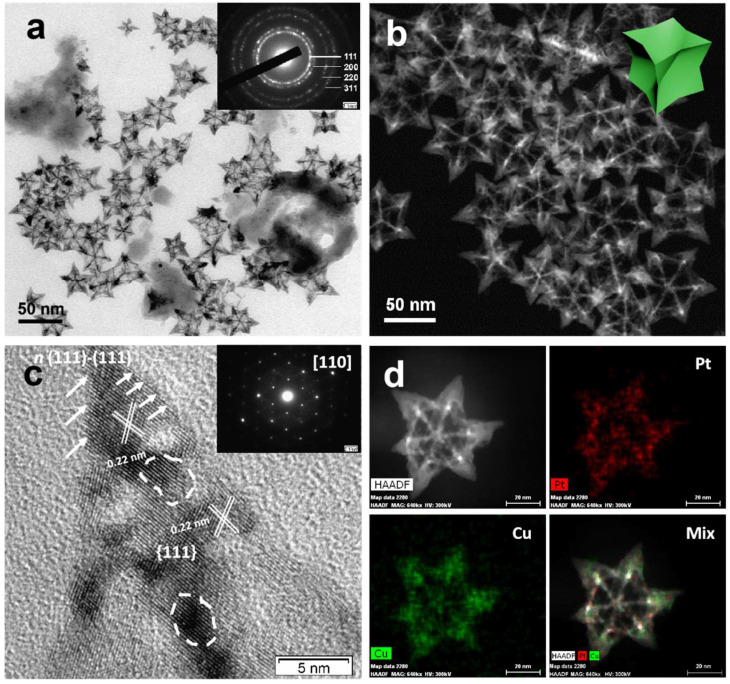
(A) TEM image of the PtCu OPCNs, the inset image shows the diffraction rings of individual CONFs; (B) The HAADF-STEM image of the as-synthesized OPCNs; (C) High resolution TEM image of one foot on the OPCNs, the white arrows show the high- index facets of n (111)-(111); The inset image shows the corresponding fast Fourier transition pattern; and (D) HAADF-STEM and EDX mapping images of an individual OPCNs.

**Figure 3:**
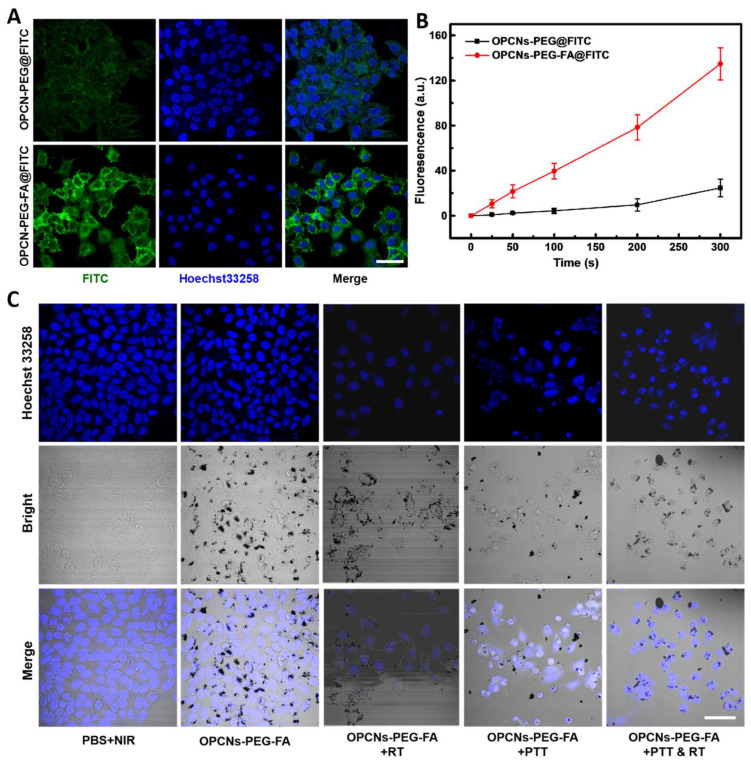
(A) CLSM images of HepG2 cells incubated with FITC alone and OPCNs-PEG- FA@FITC (200 μg/mL) nanoparticles for 6 h; (B) Fluorescence signal of OPCNs-PEG- FA@FITC in HepG2 cells (n = 5); and (C) CLSM images of HepG2 cells incubated with OPCNs-PEG-FA under different conditions. Scale bar: 50 μm.

**Figure 8:**
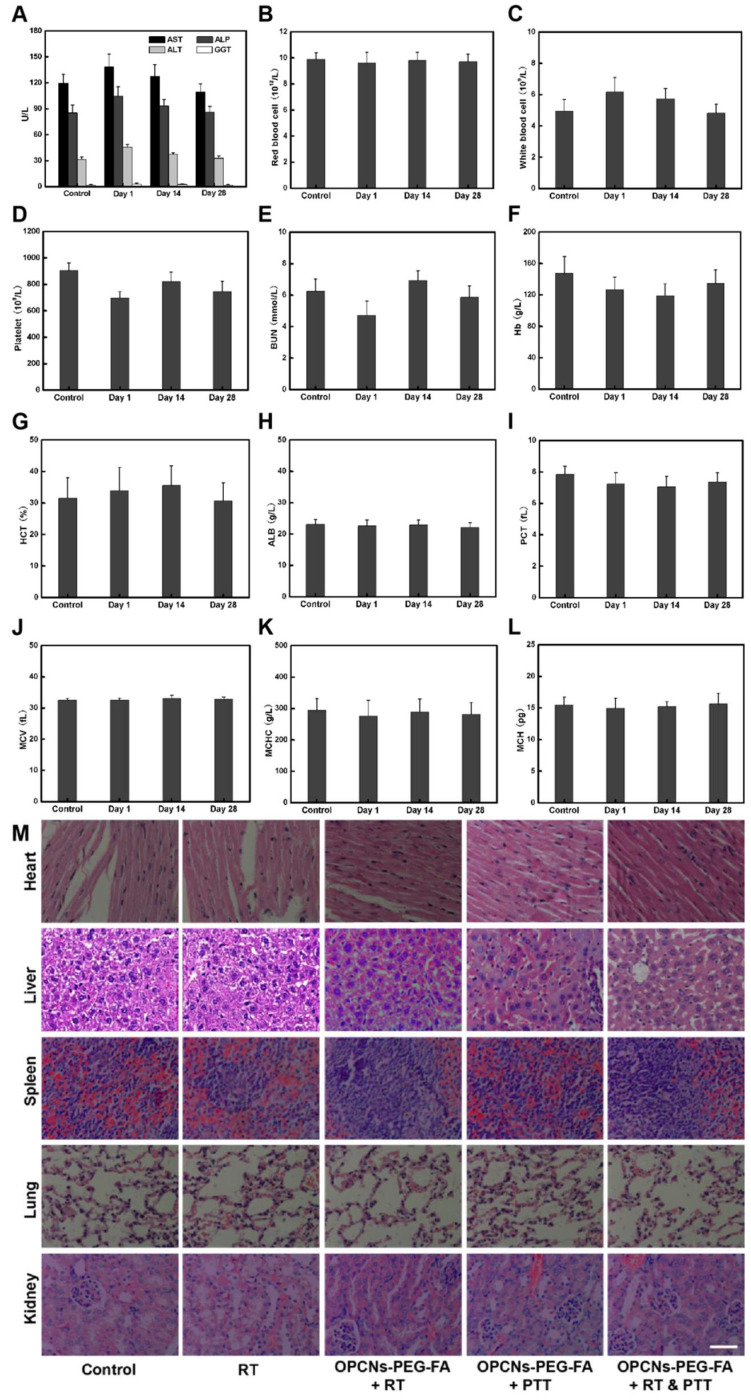
In vivo toxicology examination for mice treated with OPCNs-PEG-FA. Hematology analysis and blood biochemistry of mice determined on the 1th, 14th and 28th day (n = 5). The examined items including (A) aspartate aminotransferase (AST), alkaline phosphatase (ALP), alanine aminotransferase (ALT), and gamma- glutamyltranspetidase (GGT); (B) Red blood cell (RBC) counts; (C) White blood cell (WBC) counts; (D) Platelets (PLT) counts; (E) Blood urea nitrogen (BUN); (F) Hemoglobin (HB); (G) Hematocrit (HCT); (H) Albumin (ALB) level; (I) Plateletcrit (PCT); (J) Mean corpuscular volume (MCV); (K) Mean corpuscular hemoglobin concentration (MCHC); (L) Mean corpuscular haemoglobin (MCH); and (M) Micrographs of H&E- stained tissue sections of major organs including heart, liver, spleen, lung, and kidney of healthy mice and mice treated with OPCNs-PEG-FA on the 28th day post the last injection of nanoparticles.

